# Bionic Silk Fibroin Film Promotes Tenogenic Differentiation of Tendon Stem/Progenitor Cells by Activating Focal Adhesion Kinase

**DOI:** 10.1155/2020/8857380

**Published:** 2020-11-04

**Authors:** Kang Lu, Xiaodie Chen, Hong Tang, Mei Zhou, Gang He, Zhisong Lu, Kanglai Tang

**Affiliations:** ^1^Department of Orthopedics/Sports Medicine Center, State Key Laboratory of Trauma, Burn and Combined Injury, Southwest Hospital, Army Medical University (Third Military Medical University), Chongqing 400038, China; ^2^Institute for Clean Energy & Advanced Materials, School of Materials & Energy, Southwest University, Chongqing 400715, China

## Abstract

**Background:**

Tendon injuries are common musculoskeletal disorders in clinic. Due to the limited regeneration ability of tendons, tissue engineering technology is often used as an effective approach to treat tendon injuries. Silk fibroin (SF) films have excellent biological activities and physical properties, which is suitable for tendon regeneration. The present study is aimed at preparing a SF film with a bionic microstructure and investigating its biological effects.

**Methods:**

A SF film with a smooth surface or bionic microstructure was prepared. After seeding tendon stem/progenitor cells (TSPCs) on the surface, the cell morphology, the expression level of tenogenic genes and proteins, and the focal adhesion kinase (FAK) activation were measured to evaluate the biological effect of SF films.

**Results:**

The TSPCs on SF films with a bionic microstructure exhibited a slender cell morphology, promoted the expression of tenogenic genes and proteins, such as SCX, TNC, TNMD, and COLIA1, and activated FAK. FAK inhibitors blocked the enhanced expression of tenogenic genes and proteins.

**Conclusion:**

SF films with a bionic microstructure may serve as a scaffold, provide biophysical cues to alter the cellular adherence arrangement and cell morphology, and enhance the tenogenic gene and protein expression in TSPCs. FAK activation plays a key role during this biological response process.

## 1. Introduction

The incidence of tendon injuries has rapidly increased due to inappropriate exercise, accidents, and aging. More than 30 million tendon injuries occur every year worldwide, regardless of the number of unreported injuries [1, 2]. At present, tendon injuries are usually conservatively treated, such as physical therapy, or surgically treated, such as suturing, autologous, and allogeneic tendon transplantation [3, 4]. However, these treatments often result in scar healing formation and are associated with some disadvantages, such as low mechanical strength and tissue adhesion, which represent a commonly encountered clinical problem [5, 6]. Therefore, there is a need to develop new and practical techniques for tendon repair.

Recently, silk fibroin- (SF-) based biomaterials have been increasingly used for the repair of bone, cartilage, and other tissues due to high strength and elasticity, excellent biocompatibility, low toxicity, and easy processing [7–10]. SF can be manufactured in different forms, such as membranes, fibers, particles, and scaffolds, and is suitable for various tissues, thereby showing encouraging prospects [11–13]. SF films are of great value in the application of tendon repair due to its excellent mechanical properties and plasticity, as well as its similar structure to tendon sheath tissues, which is important for the healing process [14, 15].

The tendon structure is primarily composed of a parallel arrangement of collagen fibers with tenocytes and tendon stem/progenitor cells (TSPCs). TSPCs are multilineage-differentiating cells that can be isolated from tendons, and can be used as seeding cells to play a central role in tendon repair tissue engineering [6, 16–18]. It has been reported that SF films promote the directional differentiation of seeding cells by preparing a microstructure similar to the original tissue on its surface [19–21].

However, the effect of SF films with the microstructure of TPSCs remains unclear. The present study is aimed at investigating the effect of a novel SF film with a bionic microstructure on the biological behavior of TPSCs and exploring the preliminary mechanism. It was first found that the bionic SF film significantly changed the biological behavior of TSPCs by activating the phosphorylation of focal adhesion kinase (FAK).

## 2. Materials and Methods

### 2.1. Isolation, Identification, and Culture of TSPCs

TSPCs were isolated and identified, as previously reported by the investigators [16, 22]. Briefly, the Achilles tendon tissue was collected from three male 4-week-old Sprague Dawley (SD) rats (provided by the Animal Center of Third Military Medical University) after being euthanized. The experimental procedures were approved by the Animal Research Ethics Committee of Third Military Medical University, China. The tissues were cut into small pieces and digested with collagenase I (3 mg/mL; Sigma-Aldrich, St. Louis, MO, USA) at 37°C for 2.5 hours. Then, the single cell suspensions were obtained by passing the tissues through a cell strainer (Becton Dickinson, Franklin Lakes, NJ, USA). After cells were washed in sterile phosphate-buffer saline (PBS), these cells were centrifuged at 300 g for five minutes and resuspended in Dulbecco's modified Eagle's medium (DMEM; Gibco, Carlsbad, CA, USA). Next, these cells were cultured in DMEM supplemented with 10% fetal bovine serum, penicillin (100 U/mL)/streptomycin (100 mg/mL), and L-glutamine (2 mmol/L) (all were obtained from Invitrogen, USA) at 37°C in 5% CO_2_. After two days of culture, nonadherent cells were removed with PBS. After culturing for seven days, the TSPCs were digested in trypsin-EDTA solution (Sigma-Aldrich) and cultured as passage 0 cells. Cells from passages 3 (P3) were used for the subsequent experiments.

Immunostaining was performed to identify the stem cell characteristics of TSPCs using antibodies against CD34 (Anti-CD34 antibody, 1 : 200; ab81289), CD44 (Anti-CD44 antibody, 1 : 200; ab216647), CD3 (Anti-CD3 antibody, 1 : 200; ab135372), and CD90 (Anti-CD90/Thy1 antibody, 1 : 200; ab225). All antibodies were obtained from Abcam, Cambridge, UK.

The multipotential differentiation of TSPCs was tested in adipogenic, osteogenic, and chondrogenic induction cultures, as previously described [20]. Briefly, TSPCs were cultured in adipogenic (RASMD-90031), osteogenic (RASMD-90021), and chondrogenic (RASMD-90041) induction medium for three weeks, according to manufacturer's protocol (all were obtained from Cyagen Biosciences, Suzhou, China). The adipogenic, osteogenic, and chondrogenic differentiation results for TSPCs was assessed by Oil red O, Alizarin red, and Alcian blue staining, respectively.

TSPCs were randomly seeded on six-well culture plates (Group C), smooth SF films (Group S), and SF films with a microstructure (Group G) at a density of 5,000 cells/cm^2^. Then, these TSPCs were cultured in DMEM/F12 culture medium (Hyclone, LA, USA) supplemented with 10 U/mL of penicillin, 10 U/mL of streptomycin (Beyotime, Shanghai, China), and 10% fetal bovine blood serum (Hyclone, LA, USA). The medium was changed every three days. The SF films were tiled on the bottom of the 6-well culture plate before planting the cells. In order to investigate the effect of FAK, FAK inhibitor (7.5 *μ*M, ab144503, Abcam, Cambridge, UK) was added to the medium.

### 2.2. Preparation of the Regenerated Silk Fibroin Solution

The silk fibroin solution was prepared, as previously reported [10, 23]. Briefly, tussah silk (supplied by the State Key Laboratory of Silkworm Genome Biology, Southwest University) was boiled in 0.02 M of Na_2_CO_3_ (Aladdin Reagent Co. Shanghai, China) for 40 minutes, rinsed in dH_2_O for 20 minutes, and dried overnight at room temperature. Then, the protein extract was dissolved in 9.3 M of lithium bromide (Aladdin Reagent Co. Shanghai, China) at room temperature and heated at 50°C for five hours, followed by dialysis in water for 72 hours using a cellulose dialysis membrane (Tansoole Co. Shanghai, China). The impurities were removed by centrifugation at 8,000 r/min for 20 minutes. The resulting supernatant of the aqueous silk solution was collected, and the final concentration (4.5%wt./*v*) was determined by gravimetric analysis. The solution was stored at 4°C.

### 2.3. Preparation of PDMS Substrates

In order to prepare the smooth or microstructure polydimethylsiloxane (PDMS, provided by Wenhao Co. Ltd., Suzhou, China) substrates, the PDMS was casted on a reflective diffraction grating (provided by Wenhao Co. Ltd., Suzhou, China) with a smooth surface or microstructure surface, respectively. The microstructure had parallel grooves that were 10 *μ*m wide and 5 *μ*m deep, according to the normal tendon structure [24–26]. Round substrates of 3.5 cm in diameter were used to generate the SF films, after these were washed with 70% *v*/*v* ethanol and rinsed in distilled water.

## 3. Preparation of Silk Films

The SF films were made by casting the silk solution (3.5 mL, 4.5%wt./*v*) on the smooth or microstructure of the PDMS molds, followed by drying at 50°C for eight hours. After covering with a venting lid, the films were allowed to continue to dry overnight at 50°C and annealed for 100 minutes under a condition of 90% relative humidity, 65°C, and 20 mmHg of vacuum. Then, the dry silk films were autoclaved at 121°C for 20 minutes, and placed in 6-well plates for cell seeding.

### 3.1. Characterization of SF Films with Different Microstructures by SEM

The gold-coated SF films were examined under a scanning electron microscope (SEM; Phenom Prox, Phenom, Netherlands) at 15 kV. The cross-section of the film was used to measure the thickness of the SF films, and tiled samples were used to observe the surface morphology. ImageJ software was used to further quantify the width and spacing of the bionic microstructure of these SF films.

### 3.2. Biomechanical Test of SF Films and Normal Achilles Tendon

The biomechanical test was performed in native tendons as control (Group N), smooth SF film (Group S), and film with microstructure (Group G), as previously described [16]. In brief, Achilles tendons with bony attachments were isolated from five SD rats, and the calcaneal and tibial ends were secured, followed by the attachments of the tendon to a custom test fixture. Before testing, the SF film specimens were cut and rolled into strips (1 cm in length and 2 mm in diameter), and secured to a custom test fixture. Then, a universal testing machine (Instron E1000, MA, USA) was used to evaluate the tensile stress-strain curves of all specimens. As previously described [19], the maximum loading, ultimate stress (N/mm^2^), and breaking elongation (%) of groups N, C, and G were compared.

### 3.3. Confocal Microscopy

Confocal microscopy was used to observe the morphology of TSPCs in groups C, S, and G, as previously described [20]. After culturing for 72 hours, cells were fixed with 4% paraformaldehyde for 20 minutes, permeabilized with 0.5% Triton X-100 for five minutes, and incubated in 100 nM of rhodamine phalloidin (Yeasen Biological Technology Co, Shanghai, China) for 30 minutes. Then, the nuclei were counterstained with 100 nM of 4′6-diamidino-2-phenylindole (DAPI, Beyotime Biotech, Jiangsu, China) for five minutes. Afterwards, the TSPCs on different surfaces were examined using a laser scanning confocal microscope (Zeiss lsm780, Germany). ImageJ software [27] was used to measure the length/width ratio, major axis angles, and cell area of the cell body. The major axis angle referred to the angle between the main axis of the cell body and the vertical line [19, 20]. For each measurement, five cells per image and three images in each group were used for the data analysis.

### 3.4. Live/Dead Staining of TSPCs

After cells reached 90-100% confluency, the TSPCs in groups C, S, and G were observed and stained using a Live/Dead Cell Staining Kit (BB-4127, BestBio, Shanghai, China; 1 : 1,000). Then, these were photographed under an inverted fluorescence microscope (Olympus, IX71, Japan).

### 3.5. Cell Cytotoxicity

Cell cytotoxicity was detected using Cell Counting Kit-8 (CCK-8; C0038, Beyotime Biotech, Jiangsu, China), according to previous reports [19]. At 1, 3, 5, 7, 9, and 11 days after seeding, the TSPCs in groups C, S, and G were incubated with the 10% (*v*/*v*) CCK-8 solution in a 5% CO_2_ incubator at 37°C for two hours. Then, the absorbance at 450 nm (OD value) was detected using a microplate reader (Model 680; Bio-Rad, USA).

### 3.6. Real-Time Reverse Transcriptase Polymerase Chain Reaction (RT-PCR)

The mRNA expression levels of the tendon-related genes of Tenascin-C (TNC), Tenomodulin (TNMD), collagen type I alpha china (COLIA1), and scleraxis (SCX) were determined using real-time reverse quantitative polymerase chain reaction (RT-qPCR). After 4 and 7 days of culture, the total RNA for groups C, S, and G was extracted using the TRIzol reagent (Takara, Dalian, China). The cDNA was synthesized from 1 *μ*g of the total RNA using the Superscript III First-Strand Synthesis Kit (TaKaRa). Then, RT-qPCR was performed on the ABI Prism 7900 Sequence Detection System (PE Applied Biosystems, Foster City, CA, USA) using a SYBR Green RT-PCR kit (TaKaRa). Glyceraldehyde-3-phosphate dehydrogenase (GAPDH) was used as an internal control. The polymerase chain reaction (PCR) primers used are presented in Table 1. The delta-delta Ct method was used for the analysis of the RT-PCR data.

### 3.7. Western Blot

After 14 days of culture, the TSPCs in groups C, S, and G were lysed in lysis buffer containing a mixture of proteinase inhibitors (Thermo Fisher Scientific Inc., Rockford, IL, USA). Equal amounts of protein samples (30 *μ*g/lane) were run on sodium dodecyl sulfate–polyacrylamide gel electrophoresis and transferred onto polyvinylidene difluoride membranes. Then, these membranes were incubated with the primary antibodies overnight at 4°C. The following primary antibodies were used: anti-Tenascin C antibody (TNC, ab108930), anti-tenomodulin antibody (TNMD, ab203676), anti-SCXA antibody (SCX, ab58655), anti-Collagen I antibody (COLIA1, ab6308), anti-FAK (FAK, ab40794), anti-FAK (phosphoY397, pFAK, ab81298), and anti-GAPDH antibody (GAPDH, ab8245). All primary antibodies were purchased from Abcam (Cambridge, UK). GAPDH was used as a loading control. Then, these membranes were incubated with the secondary antibodies (10285-1-AP, 1 : 2,000; Proteintech, Wuhan, China) for two hours at room temperature. The protein bands were visualized using the enhanced chemiluminescence detection kit (GE Healthcare, Wuxi, China).

### 3.8. Statistical Analysis

The statistical analysis was performed using SPSS 22.0. If not stated otherwise, all experiments were performed in triplicate, and the data were presented as mean ± standard deviation. The quantitative data was analyzed using analysis of variance (ANOVA) and post hoc multiple comparison by Tukey's test. A *P* value of <0.05 was considered statistically significant. The significance level was presented as ^∗^*P* < 0.05 or ^∗∗^*P* < 0.01.

## 4. Results

### 4.1. Isolation and Identification of Rat TSPCs

**Figure 1:**
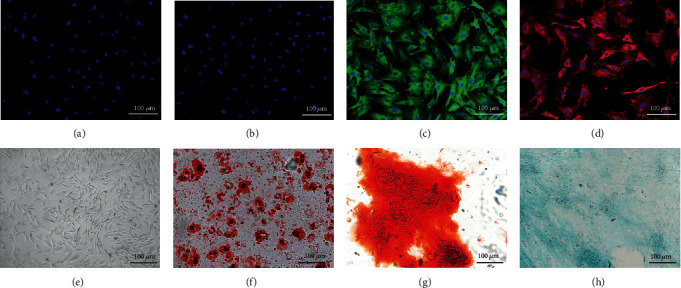
Isolation and identification of TSPCs: (a–d) immunofluorescence staining of CD3 (a), CD34 (b), CD44 (c), and CD90 (d); (e) cell morphology of TSPCs on the tissue culture plate under a light microscope; (f–h) Oil red O staining, Alizarin red staining, and Alcian blue staining of TSPCs after the induction of adipogenesis, osteogenesis, and chondrogenesis.

The immunostaining was performed to characterize the newly isolated rat TSPCs using antibodies against leukocyte marker CD3, hematopoietic stem cell marker CD34, and stem cell markers CD44 and CD90 [28, 29]. TSPCs were negative for hematopoietic stem cell marker CD34 and leukocyte marker CD3, but positive for stem cell markers CD44 and CD90 (Figures 1(a)–1(d)). These results indicate that TSPCs exhibit the characteristic feature of stem cells and that these cells were derived from the tendon, but not from the blood [30]. The TSPCs presented with a spindle-shape and fibroblast-like morphology at low density. At a confluence of 80%-90%, these TSPCs presented with a pebble-like morphology with a tight colony formation (Figure 1(e)).

The Oil red O staining revealed that after adipogenic induction, round orange droplets were present within the cytoplasm of TSPCs, indicating that lipid droplets were formed in TSPCs (Figure 1(f)). The Alizarin red staining revealed that the extracellular deposited calcium salt was stained red, indicating that an osteo-matrix formed around the TSPCs after osteogenic induction (Figure 1(g)). The Alcian blue staining revealed that acidic glycosaminoglycans were stained blue, indicating that an extracellular cartilage matrix formed after chondrogenic induction (Figure 1(h)).

### 4.2. Characterization of SF Films by SEM and the Biomechanical Test

**Figure 2:**
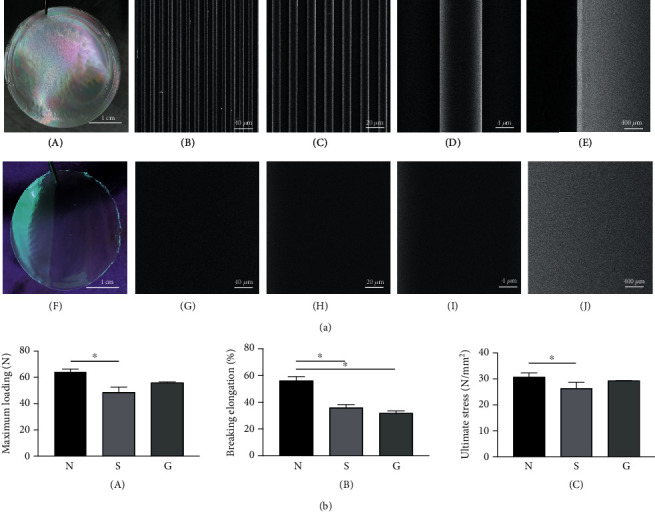
SEM and mechanical test. (a) Characterization of SF films: (A) the photograph of the SF film with a microstructure; (B–E) the SEM images of the SF film with a microstructure; (F) the photograph of the smooth SF film; (G–J) the SEM images of the smooth SF film; magnifications: 2,000x (B, G), 10,000x (C, H), 20,000x (D, I), and 100,000x (E, J). (b) Mechanical properties of the native tendon (group N), smooth SF film (group S), and SF film with a microstructure (group G): (A) maximum loading, (B) breaking elongation, (C) ultimate stress; ^∗^*P* < 0.05.

Under a scanning electron microscope, the microstructure on the surface of the SF film was clear at 2,000-100,000x magnification (Figure 2(a), A–J). The smooth SF film surface had no uneven structure, and the microstructure of the SF film was clear, with a spacing of 10 *μ*m.

The biomechanical test results revealed that SF films had good mechanical properties. As shown in Figure 2(b), A–C, the smooth SF film (group S) had a significantly lower maximum loading (*P* = 0.023) and ultimate stress (*P* = 0.037), when compared to the native tendon (group N). However, there was no significant difference in maximum loading and ultimate stress between group N and group G. Furthermore, group S (*P* = 0.012) and group G (*P* = 0.035) had a lower breaking elongation rate, when compared to group N.

### 4.3. The Morphology of TSPCs

**Figure 3:**
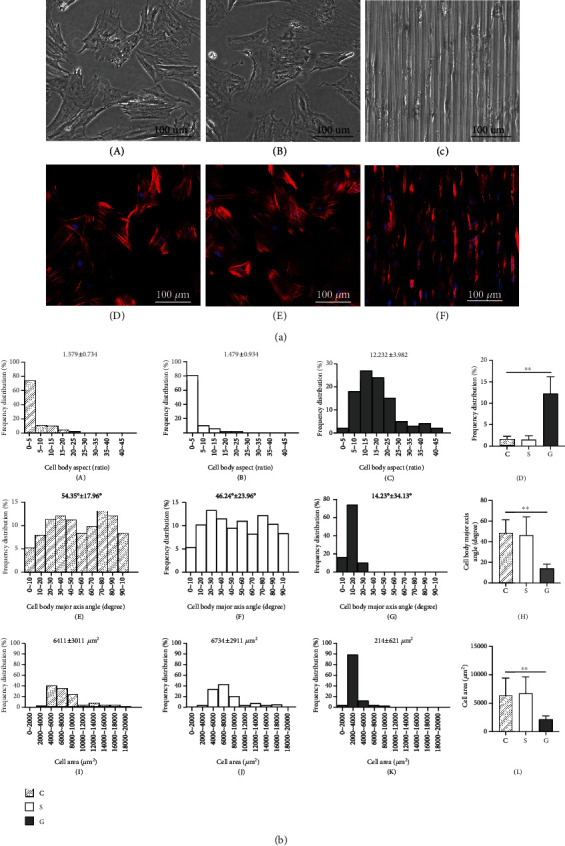
The cell morphology of TSPCs on different matrix surfaces. (a) Cell morphology observation: (A–C) cell morphology under a light microscope; (D–F) the morphology of TSPCs under a confocal laser scanning microscope. The nuclei were stained blue; the cytoskeletons were stained red; (A, D) TSPCs in the cell culture plate; (B, E) TSPCs on the smooth SF film; (C, F) TSPCs on the SF film with a microstructure. (b) Analysis of cell morphology: (A–D) cell body aspects; (E–H) cell body major axis angle (I–L) cell area; group C: TSPCs on the cell culture plate; group S: TSPCs on the smooth SF film; group G: TSPCs on the SF film with a microstructure; ^∗∗^*P* < 0.01.

TSPCs were seeded on the cell culture plate (group C), smooth SF film (group S), and SF film with a microstructure (group G). These TSPCs presented with a polygonal shape in group C and group S, but exhibited an elongated cell morphology in group G (Figure 3(a), A–C). Similar morphological changes were observed in the cytoskeleton and DAPI staining images of TSPCs (Figure 3(a), D–F). In order to further quantify the morphological changes of TSPCs in the different groups, the length/width ratio of the cell body, cell body major axis angle, and total cell area were analyzed. Compared with group C, the TSPCs in group G had a greater length/width ratio (Figure 3(b), A–D, *P* = 0.0027), a less cell body axis angle (Figure 3(b), E–H, *P* = 0.0031), and a reduced cell area (Figure 3(b), I–L, *P* = 0.0039), suggesting that the seeded cells on SF films with a bionic microstructure had different oriented arrangements with a slender cell morphology. Therefore, SF films with a microstructure can induce the orientation of cells and alter the cell adhesion morphology.

### 4.4. Cytotoxicity of SF Films

**Figure 4:**
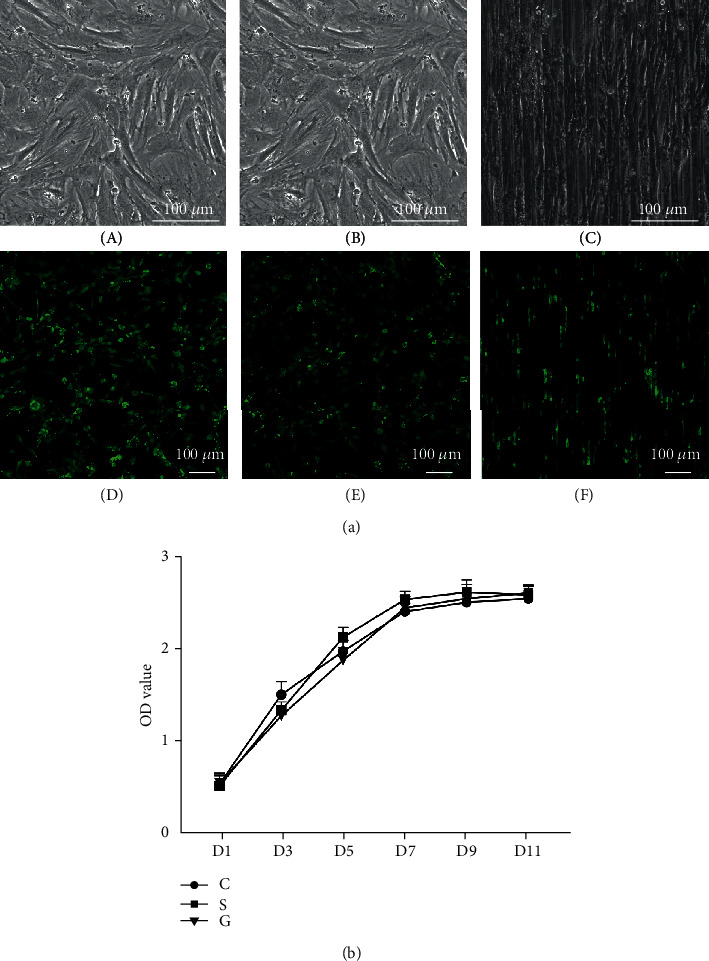
Live/dead cell staining and CCK-8 assay. (a) (A–C) Optical photomicrographs after TSPCs reached 90-100% confluence; (D–F) live/dead staining of TSPCs on the tissue culture plate (D), SF film with a smooth surface (E), and SF film with microstructure (F). (b) The CCK-8 curve of the different groups; group C: TSPCs on the cell culture plate; group S: TSPCs on the smooth SF film; group G: TSPCs on the SF film with a microstructure.

The TSPCs reached 90-100% confluence in all groups on day seven (Figure 4(a), A–C). The OD value reached a plateau from day seven (Figure 4(b)). On day seven, the live/dead staining results revealed that TSPCs on the tissue culture plate (Figure 4(a), D), smooth SF film (Figure 4(a), E), and SF film with a bionic microstructure (Figure 4(a), F) maintained its good viability. There was no significant difference in cell viability among the three groups (Figure 4(b)), indicating that the SF film biomaterial was not cytotoxic and had good biocompatibility.

### 4.5. Tenogenic Differentiation of TSPC on SF Films with a Bionic Microstructure

**Figure 5:**
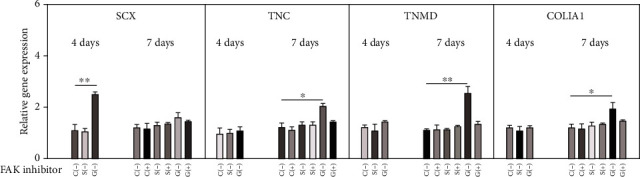
The relative mRNA expression of SCX, TNC, TNMD, and COLIA1 on days 4 and 7, with or without the FAK inhibitor. *n* = 3, ^∗^*P* < 0.05, and ^∗∗^*P* < 0.01.

The PCR results revealed that the expression of tenogenic markers SCX, TNC, TNMD, and COLIA1 was significantly higher in group G, when compared to group C and group S. As shown in Figure 5, in group G without the FAK inhibitor, the expression of transcription factor SCX significantly increased on day four (*P* = 0.008), and tenogenic differentiation specific markers TNC (*P* = 0.041), TNMD (*P* = 0.006), and COLIA1 (*P* = 0.023) significantly increased on day seven. However, the FAK inhibitors blocked the increase in gene expression levels of SCX, TNC, TNMD, and COLIA1 in group G.

**Figure 6:**
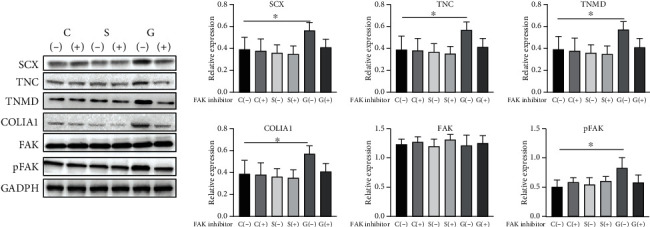
Western blot analysis of the protein expression level of SCX, TNC, TNMD, COLIA1, FAK, and pFAK, and GAPDH was used as the loading control; *n* = 3, ^∗^*P* < 0.05, and ^∗∗^*P* < 0.01.

The tenogenic protein expression of SCX, TNC, TNMD, and COLIA1 is presented in Figure 6. In group G without the FAK inhibitor, the expression of SCX (*P* = 0.038), TNC (*P* = 0.041), TNMD (*P* = 0.004), and COLIA1 (*P* = 0.0071) significantly increased. These results suggest that SF films with a microstructure promote the tenogenic differentiation of TSPCs *in vitro*. However, the FAK inhibitors blocked the increase in gene expression levels of SCX, TNC, TNMD, and COLIA1 in group G.

### 4.6. The Activation of FAK on SF Films with a Microstructure

As shown in Figure 6, there was no significant difference in FAK expression among groups C, S, and G, but the expression of pFAK in group G without the FAK inhibitor (*P* = 0.092) significantly increased. This revealed that SF films with a microstructure promote the phosphorylation of FAK in TSPCs.

## 5. Discussion

SF films have been widely used in soft tissue engineering, such as the cartilage and cornea, in recent years [9, 10, 31, 32]. SF films can be used for tendon repair due to its high mechanical property. Most importantly, the intrinsic *β*-sheet of SF is similar to tropocollagen, and the *α*-helix and irregular curl are similar to the noncollagenous structures in tendons [11, 13, 15, 33]. In the present study, SF films with a bionic microstructure had good mechanical properties and can be used as a scaffold material for tendon tissue engineering [34].

Previous studies have shown that the biological materials in tissue repair not only provide good biological support but also guide the biological behavior of seeding cells [34–36]. Therefore, growth factors and active groups, such as the BMP-2 and RGD sequence, are usually added to the biological material, in order to change the biological behavior of seeding cells. However, there are some disadvantages associated with this method, which include the poor mechanical properties of the biological material and the inactivation of the growth factor in the biological material [37–39]. In the present study, the bionic microstructure on the surface of SF films was prepared to enhance its biological effect. The TSPCs on SF films with a bionic microstructure presented with an oriented spatial arrangement, a slender cell morphology, and enhanced expression of tenogenic differentiation related genes and proteins. Compared to other biomaterials [39], the SF films with a microstructure prepared in the present study changed the differentiation tendency of TSPCs by providing physical stimulation signals [40], and the biological activity was more stable and precise, suggesting that this has a higher application value for tendon repair.

FAK activation plays a key role in changes in seeding cell behaviors induced by the surface of the material [41–43]. In the present study, it was found that FAK activation mainly occurred in TSPCs on SF films with a microstructure, accompanied by an obvious elongated morphology. These findings suggest that on the surface of SF films with a microstructure, FAK phosphorylation activation and TSPC morphological changes were strongly associated with each other. Cell morphology changes may reset the arrangement of actin in TSPCs and alter the stress direction. This mechanical signal may transmit into cells through integrin molecules, subsequently activating FAK and its downstream signaling pathways, and eventually passing into the nucleus to change the cell fate [44, 45]. In the present study, the physical signals provided by the bionic SF film increased the expression of tendon-related genes and proteins in TSPCs, but this effect was blocked by FAK inhibitors. These findings suggest that bionic SF films activate the phosphorylation of FAK through the changes in cell morphology and spatial arrangement and ultimately alter the differentiation fate of TSPCs. The biological effect of bionic SF films provides a theoretical basis for its application in tendon tissue engineering. However, the specific molecular mechanisms involved in this process require further basic research.

## 6. Conclusion

SF films with a bionic microstructure can serve as a valuable scaffold for tendon repair, since these induce TSPCs to be an oriented alignment with slender cell morphology. Furthermore, SF films with a bionic microstructure enhance the expression of tenogenic genes in TSPCs by activating the FAK phosphorylation.

## Figures and Tables

**Table 1:** Primers used in qPCR analysis.

Gene name		Annealing temperature (°C)	PCR product size (bp)
GAPDH	F: GACTTCAACAGCAACTCCCAC	60	125
	R: TCCACCACCCTGTTGCTGTA		
Tenomodulin	F: TGTACTGGATCAATCCCACTCT	60	115
	R: GCTCATTCTGGTCAATCCCCT		
Scleraxis	F: CCTTCTGCCTCAGCAACCAG	60	156
	R: GGTCCAAAGTGGGGCTCTCCGTGACT		
Tenascin-C	F: CAAGGGAGACAAGGAGAGTGAT	60	159
	R: AGGCTGTAGTTGAGGCGGTAAC		
COLIA1	F: GGCGGCCAGGGCTCCGACCC	60	320
	R: AATTCCTGGTCTGGGGCACC		

## Data Availability

The datasets generated and analyzed during the present study are available from the corresponding author on reasonable request.
