# Corrigendum to “Bionic Silk Fibroin Film Promotes Tenogenic Differentiation of Tendon Stem/Progenitor Cells by Activating Focal Adhesion Kinase”

**DOI:** 10.1155/2021/6317062

**Published:** 2021-03-02

**Authors:** Kang Lu, Xiaodie Chen, Hong Tang, Mei Zhou, Gang He, Zhisong Lu, Kanglai Tang

**Affiliations:** ^1^Department of Orthopedics/Sports Medicine Center, State Key Laboratory of Trauma, Burn and Combined Injury, Southwest Hospital, Army Medical University (Third Military Medical University), Chongqing 400038, China; ^2^Institute for Clean Energy & Advanced Materials, School of Materials & Energy, Southwest University, Chongqing 400715, China

In the article titled “Bionic Silk Fibroin Film Promotes Tenogenic Differentiation of Tendon Stem/Progenitor Cells by Activating Focal Adhesion Kinase” [1], the authors identified that incorrect figures were included for Figures 3 and 4 due to a typesetting error. The authors confirm that this does not affect the results of the article, and the corrected Figures 3 and 4 are as follows:

## Figures and Tables

**Figure 1:**
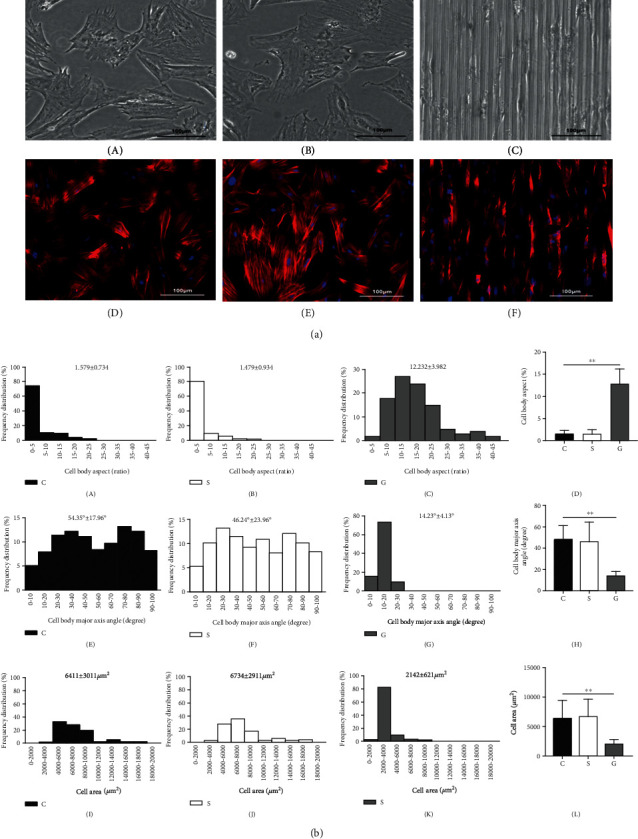
The cell morphology of TSPCs on different matrix surfaces. (a) Cell morphology observation: (A–C) cell morphology under a light microscope; (D–F) the morphology of TSPCs under a confocal laser scanning microscope. The nuclei were stained blue; the cytoskeletons were stained red; (A, D) TSPCs in the cell culture plate; (B, E) TSPCs on the smooth SF film; (C, F) TSPCs on the SF film with a microstructure. (b) Analysis of cell morphology: (A–D) cell body aspects; (E–H) cell body major axis angle (I–L) cell area; group C: TSPCs on the cell culture plate; group S: TSPCs on the smooth SF film; group G: TSPCs on the SF film with a microstructure; ^∗∗^*P* < 0.01.

**Figure 2:**
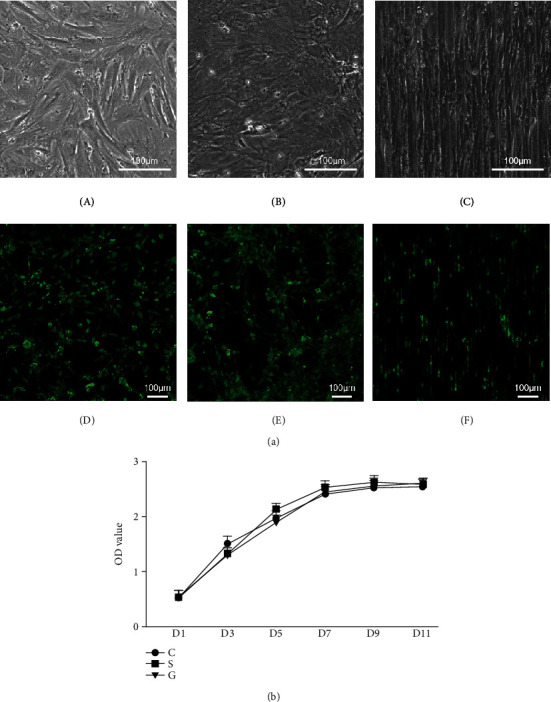
Live/dead cell staining and CCK-8 assay. (a) (A–C) Optical photomicrographs after TSPCs reached 90-100% confluence; (D–F) live/dead staining of TSPCs on the tissue culture plate (D), SF film with a smooth surface (E), and SF film with microstructure (F). (b) The CCK-8 curve of the different groups; group C: TSPCs on the cell culture plate; group S: TSPCs on the smooth SF film; group G: TSPCs on the SF film with a microstructure.
